# Prolonged Combination Therapy is More Effective than Monotherapy in Management of Chronic Hepatitis B Patients With Sustained Virological Response: An Experience From a ‘Real-World’ Clinical Setting

**DOI:** 10.5812/ircmj.7788

**Published:** 2013-12-05

**Authors:** En Qiang Chen, Lang Bai, Lan Lan Chen, Tao You Zhou, Ling Yao Du, Hong Tang

**Affiliations:** 1 Center of Infectious Diseases, West China Hospital, Sichuan University, China; 2 Division of Infectious Diseases, State Key Laboratory of Biotherapy, Sichuan University, China

**Keywords:** Hepatitis B, Chronic, Combined Modality Therapy, Hepatitis Be Antigens

## Abstract

**Background:**

Little is known about the duration of combination therapy for patients with chronic hepatitis B (CHB) and suboptimal response to nucleos(t)ide analogues(NAs) monotherapy.

**Objectives:**

This study aimed to assess whether monotherapy could be used for treatment of CHB patients, who poorly responded to Adefovir Dipivoxil (ADV) but obtained good responses after at least 12-month lamivudine (LAM) or telbivudine (LdT) add-on therapy.

**Patients and Methods:**

Forty-five patients were enrolled, and the baseline time-point was determined according to enrollment data. Twenty-six patients chose to continue combination therapy (LAM+ADV or LdT+ADV, Group A) and 19 patients switched to single-drug maintenance therapy (LAM or LdT or ADV, Group B).

**Results:**

There were no signiﬁcant differences between two groups in baseline characteristics (P > 0.05). At 12th month, sustained virological response rate was greater in group A compared to group B (96.2% vs. 47.4%, P < 0.001), and the rates of NAs-associated resistance were 0% in group A and 15.8% in group B. Alanine aminotransferase normalization rate was also signiﬁcantly higher in group A compared with group B (92.3% vs. 36.8%, P < 0.001). Among hepatitis positive patients with Be antigen (HBeAg)-, 40% (4/10) in group A and 9.1% (1/11) in group B achieved HBeAg seroconversion at the 12th month. Of patients in group B with positive-HBeAg before the previous combination therapy and detectable HBV DNA at 6 months of previous combination therapy were associated with high risks of viral relapse after switching to single-drug maintenance therapy.

**Conclusions:**

Prematurely switching to single-drug maintenance therapy would be resulted in viral relapse, and prolonged combination therapy was effective to maintain sustained responses for patients with initial suboptimal response to ADV.

## 1. Background

About a quarter of the world population, more than 2 billion people, have been infected with hepatitis B virus (HBV), including 350 million people with chronic hepatitis B (CHB) (1, 2). According to the report from World Health Organization, HBV infection is highly endemic in the Asia pacific regions (3-6), and 15 to 25% of CHB patients would progress to life-threatening liver diseases including cirrhosis and hepatocellular carcinoma (3, 5).

Although completely eradication of HBV is seldom achieved by current antiviral treatments, but long-term suppression of HBV DNA to undetectable levels with antiviral therapy has been shown to significantly reduce liver disease progression (6, 7). At present, several nucleos(t)ide analogues (NAs) are available for the treatment of CHB (8, 9), and the treatment usually requires a long time to get the responses. Recently, high rates of suboptimal viral response and resistance have diminished the long-term clinical benefits of antiviral therapy (8, 10). Because of limited choice of antiviral agents, more attention has gradually been drawn to the strategy of combination therapy (11). In recent years, increasing evidence suggests that combination therapy could effectively suppress viral replication and significantly delay or prevent the emergence of drug resistance (11-13), and combination therapy has become a potentially attractive therapeutic option in clinical practices (14, 15). Compared to other antiretroviral therapies, the experience of combination antiviral therapy in CHB patients is relatively limited, and both the duration and long-term effectiveness of combination therapy remain to be confirmed (16). Though some evidence suggested that the rates of viral rebound and/or recurrence were high with the cessation of antiviral therapy (17-19), it is still unclear whether switching from combination therapy to single-drug maintenance therapy would lead to high viral rebound and/or accelerated disease progression for patients who already had achieved sustained virological response. In addition to long-term sustained suppression of viral replication of CHB, how to limit the medication regimen complexity and decrease the cost of drugs also have been widely concerned (16, 20, 21), because it is mainly associated with CHB patients' compliance of antiviral therapy.

In recent years, a strong doubt has been raised about the necessity of long-term combination therapy on CHB patient, which just poorly responded to single NAs but already showed good responses after receiving a certain dose of combination therapy; and this doubt also plagued our clinicians and patients in ‘real-world’ clinical practices.

## 2. Objectives

The aim of this study was to assess whether switching-to monotherapy could be used for the treatment of CHB patients, who poorly responded to Adefovir Dipivoxil (ADV) but had good responses after receiving at least a 12-month lamivudine (LAM) or telbivudine (LdT) add-on therapy.

## 3. Patients and Methods

### 3.1. Study Design

In our previous study (22), the efficacy of LAM + ADV or LdT + ADV combination therapy on 72 CHB patients with suboptimal response to ADV (serum HBV DNA levels > 3 log10 copies/mL by PCR assays after ≥ 12 months of initial ADV monotherapy) were assessed; and after 1-year combination therapy, a total of 61 patients had a normal level of alanine aminotransferase (ALT) and sustained virological response.

This is a prospective, non-randomized cohort study from February 2011 to April 2012, to assess whether switching to single-drug maintenance therapy could be used as a treatment of CHB patients , who poorly responded to ADV but obtained good responses after receiving at least 12 months of LAM + ADV or LdT + ADV combination therapies. This study was conducted in Chengdu, in west China; and all the 61 candidates were recruited in present ‘real-world’ study (ChiCTR.org identifier: ChiCTR-ONRC-12001921). Of 61 candidates, 16 patients withdrew from this study for various reasons, and 45 patients successfully were enrolled: 26 patients involved in continuous combination treatment (LAM + ADV or LdT + ADV, Group A) and 19 patients in single-drug maintenance treatment (LAM or LdT or ADV, Group B) according to their personal choices.

All included patients were prospectively followed-up every 3 months, and baseline time-point of those patients was determined according to their enrollment date.

The inclusion criteria in this study were as follows: 1) lack of co-infection with hepatitis C virus or human immunodeﬁciency virus; 2) lack of other causes of liver disease, such as autoimmune hepatitis, and primary biliary cirrhosis; 3) no evidence of advanced liver diseases, such as decompensated cirrhosis, severe hepatitis, and hepatic carcinoma. Patients were excluded if they had poor compliance or no availability of detailed laboratory test results.

The study was conformed to the ethical guidelines of the 1975 Declaration of Helsinki. The approval of this study was obtained from the Ethics Committee of West China Hospital of Sichuan University, and written informed consent was obtained from participants.

### 3.2. Laboratory Tests

In present study, biochemical tests were performed using standard procedures (Olympus AU5400, Olympus Corporation, Tokyo, Japan), the upper limit of normal ALT was deﬁned as 55 IU/L for men and 38 IU/L for women. Serological markers, including hepatitis B surface antigen (HBsAg), hepatitis B e antigen (HBeAg) and anti hepatitis B e antibody (Anti-HBe) were evaluated by electrochemiluminescence immunoassay (Elecsys; Roche Diagnostics, China). Serum HBV DNA extraction and real-time PCR quantification were performed by fully automatic COBAS AmpliPrep/COBAS TaqMan 48 system (CAP-CTM; Roche, Branchburg, NJ), with a lower detection limit of 70 copies/mL. HBV genotype and drug-resistance mutations to NAs were detected by pyrosequencing.

### 3.3. Definition

In present study, treatment positive responses were calculated 12 months after study enrollment. Viral relapse was defined as HBV DNA detected by PCR after achieving undetectability of HBV DNA (< 70 copies/mL), and confirmed in two consecutive times at least one month apart. Sustained viral response (SVR) was defined as a persistent of undetectable serum HBV DNA at month 12. HBeAg serological response was deﬁned as a confirmed seroconversion of HBeAg to Anti-HBe in HBeAg-positive patients at baseline. Combined response (CR) was defined as a combination of normal serum ALT level and undetectable serum HBV DNA at month 12, regardless of HBeAg status at baseline in present study.

### 3.4. Statistical Analysis

Quantitative variables were expressed as mean, 95% confidence interval (95% CI), median and interquartile range (IQR). Categorical variables were presented as number and percentages. By considering the lower sample size, the comparisons between quantitative variables were performed using T-test or Mann-Whitney U test with rootstrap resampling; and the evaluation of qualitative variables were performed using Chi-square test or Fisher’s exact test. The statistical analysis was carried using the SPSS 19.0 (SPSS Inc., Chicago, IL, USA). P-value less than 0.05 (two-tailed) indicating significant difference. The values of PPV and NPV were also calculated to determine the reliability of predictors of SVR in single-drug maintenance therapy.

## 4. Result

### 4.1. Patient Characteristics

**Figure 1.**
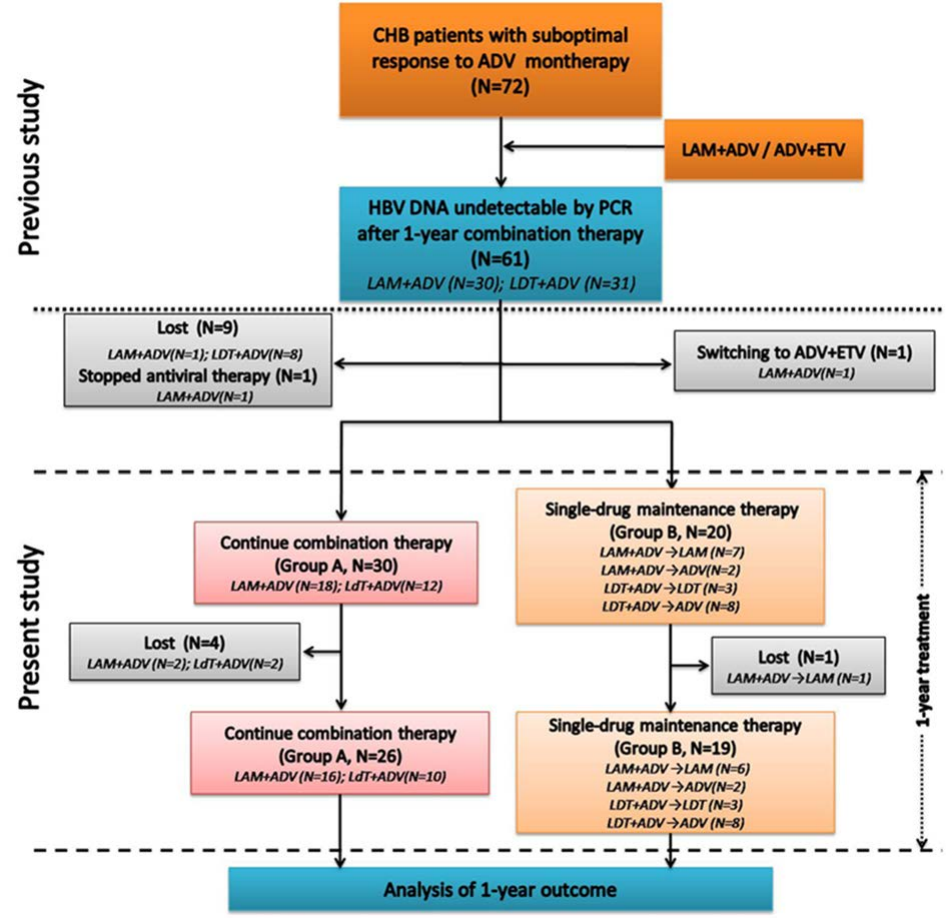
Screening and Follow-up Flow Chart

A total number of 45 patients were finally included in the study (Figure 1), including 10 HBeAg-positive patients in group A and 11 HBeAg-positive patients in group B. The baseline characteristics of CHB patients between two groups were summarized in Table 1, and the standard demographic and laboratory characteristics were similar in two groups (P > 0.05). 

**Table1.** Baseline Clinical Features of the Patients in two Groups

Characteristic	Continue Combination Therapy (Group A)	Single-Drug Maintenance Therapy (Group B)	*P*-value
**Sample size, No.**	26	19	
**Male, No. (%)**	16 (61.54)	11 (57.9)	0.805
**Age, y**			
Mean (95%CI)	34.4 (31.6-36.8)	35.3 (31.8-38.4)	0.674
Median (IQR)	35.0 (9.25)	35.0 (12)	
**Weight**			
Mean (95%CI)	59.3 (57.0-63.2)	55.8 (51.5-58.2)	0.109
Median (IQR)	60.0 (11.25)	55.0 (8.0)	
**Family history of HBV infection, No. (%)**	14 (53.8)	8 (42.1)	0.436
**Positive HBeAg, No.(%)**	10 (38.5)	11 (57.9)	0.197
**Duration of past combination therapy, mo**			
Mean (95%CI)	15.4 (14.2-17.2)	13.5 (12.8-14.0)	0.717
Median (IQR)	14.0 (3.0)	13.0 (3.0)	
**Duration of past ADV monotherapy, – mo**			
Mean (95%CI)	13.6 (12.9-14.0)	17.7 (15.8-21.0)	0.109
Median (IQR)	15.0 (4.5)	16.0 (11.0)	
**Past treatment with LAM+ADV/LdT+ADV, No. (%)**	16 (61.5)/10 (38.5)	8 (42.1)/11 (57.9)	0.197

### 4.2. Treatment Response

The treatment outcomes of two groups are summarized in Table 2. At the beginning, all patients had normal ALT and undetceable HBV DNA. After 12 months of therapy, 92.3% (24/26) patients showed normal persistent ALT level and 96.2% (25/26) had SVR in group A, while only 36.8% (7/19) patients had normal persistent ALT level and 47.4% (9/19) had SVR in group B; and the difference in either persistent ALT normalization or SVR between two groups were statistically significant (both P < 0.001). With respect to the combined response (CR), the rate of CR in group A was also significantly greater than group B (92.3% (24/26) *vs. *36.8% (7/19), P < 0.001). 

Among patients who were HBeAg-positive at baseline, 40% (4/10) of patients in group A and 9.1% (1/11) of patients in group B were HBeAg seroconversion at month 12, respectively; and the rate of HBeAg seroconversion between two groups was similar (P = 0.149).

### 4.3. Viral Relapse and Resistance

In total, 11 patients experienced VB during 12 months of observation, including 1 patient (1/26, 3.8%) in group A and 10 patients (10/19, 52.6%) in group B. Of the 10 patients in group B, 4 patients had HBV DNA level between 1000 and 100,000 copies/mL, which did not meet the minimum viral load required for analytical detecting of NAs-resistant strains.

Among 7 patients, by using genotypic drug-resistant analysis at month 12, 1 patient in group A and 3 patients in group B did not have any mutation conferring resistance to LAM, ADV, or LdT. While the other 3 patients (15.8%, 3/19) in group B showed NAs-associated resistance (Table 2), including1 patient with rtM204V+rtL180M combined mutation (LAM-resistance), and 2 patients with rtA181T+rtN236T combined mutations (ADV-resistance). 

**Table 2.** Virological, Biochemical and Serological Responses at Month 12

Responses	Continue Combination Therapy (Group A)	Single-drug Maintenance Therapy(Group B)	P-value
**Sample Size, No.**	26	19	
**Sustained viral response**			
**Patients, No. (%)**	25 (96.2)	9 (47.4)	< 0.001
**Persistent ALT normalization**			
**Patients, No. (%)**	24 (92.3)	7 (36.8%)	< 0.001
**HBeAg seroconversion** ^**a**^			
**Patients, No. (%)**	4/10 (40)	1/11 (9.1)	0.149
**Combined response**			
**Patients, No. (%)**	24/26 (92.3)	7/19 (36.8)	<0.001
**NAs-associated resistance**			
**Patients, No. (%)** ^**b**^	0 (0%)	3 (15.8%)	

^a^for HBeAg positive patients enrolled in this study.

^b^because of small sample size, the difference between two groups was not analyzed statistically.

### 4.4. Safety

The majority of patients were well tolerated in two groups. The increase in serum creatinine level was not observed in both groups. Among patients in group A, 1 patient with LDT+ADV showed a serum creatine kinase slightly increase (260 U/L, normal range: 38 - 174 U/L) but had normalized serum creatine kinase MB, and 1 patient with LAM+ADV showed a decline in the quality and duration of sex life; and there was no discontinuation due to these adverse events. Among patients in group B, 1 patient with ADV reported hair loss, and 2 patients with ADV reported liver cirrhosis by abdominal ultrasonography. In both groups, no decompensated cirrhosis and hepatocellular carcinoma were reported.

### 4.5. Associated Factors to SVR in Patients Switching to Monotherapy

The clinical features of SVR and non-SVR patients at month 12 in group B were studied. As shown in Table 3, HBeAg positivity prior the past combination therapy (P = 0.02) and undetectable serum HBV DNA at month 6 (P = 0.033) were associated with probability of achieving SVR in patients in group B. While the baseline of HBeAg in this study (P = 0.141), before ADV monotherapy (P = 0.682), and the type of past combination therapy (P = 0.628) were not related to SVR for patients in group B who switched to single-drug maintenance therapy in this study. 

**Figure 2.**
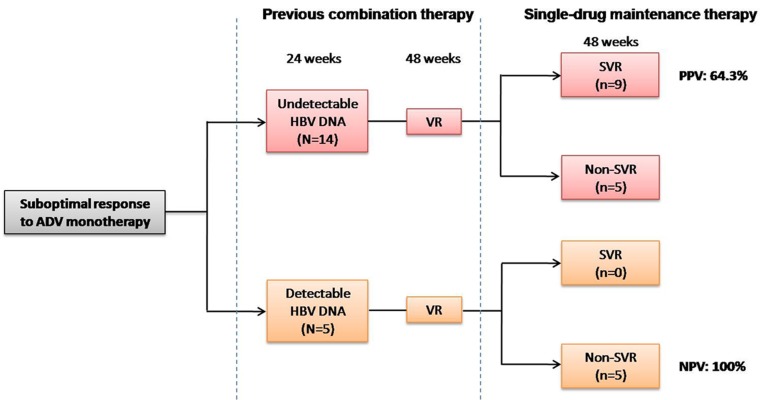
Sustained Viral Response (SVR) Distributions at week 48 in Group B According to Serum Viral Level at Week 24 of Previous Combination Therapy

As showed in Figure 2, the serum HBV DNA level at week 24 of past combination therapy prior to enrollment, had a positive predictive value (PPV) of 64.3% and a negative predictive value (NPV) of 100% for SVR at month 12 in group B patients, and these findings indicated that detectable serum HBV DNA level at week 24 of past combination therapy was associated with an extremely lower probability of maintaining SVR after switching to single-drug maintenance therapy. 

**Table 3.** Comparison of Clinical Features in SVR and non-SVR Patients at Month 12 in Group B

Characteristic	SVR	Non-SVR	P-value
**Sample size, No.**	9	10	
**Male sex, No. (%)**	6 (66.7)	5 (50.0)	0.650
**Age, y**			
Mean (95%CI)	35.6 (31.4-39.8)	35.0 (29.2-37.9)	0.804
Median (IQR)	35.0 (14.0)	32.0 (11.8)	
**Weight, kg**			
Mean (95%CI)	55.7 (51.2-60.3)	55.9 (51.4-59.8)	0.935
Median (IQR)	53.0 (9.0)	55.5 (8.0)	
**Family history of HBV infection, No. (%)**	3 (33.3)	5 (50)	0.650
**Positive-HBeAg at baseline of this study, No. (%)**	5 (55.6)	9 (90)	0.141
**Positive-HBeAg prior to past combination therapy,** **No. (%)** ^*^	3 (33.3)	9 (90)	0.02
**Duration of past ADV monotherapy, mo**			
Mean (95%CI)	18.2 (15.5-22.4)	17.2 (13.7-20.9)	0.674
Median (IQR)	16.0 (9.0)	14.5 (12.3)	
**Past combination therapy prior to enrollment**			
**LAM+ADV/LdT+ADV**	2/7	4/6	0.628
**Undetectable HBV DNA at month 3, No. (%) **	5 (55.6)	4 (40)	0.656
**Undetectable HBV DNA at month 6, No. (%)**	9 (100)	5 (50)	0.033

## 5. Discussion

This prospective, cohort study is the first ‘real-world’ assessment of different post-processing strategies of combination therapy in patients with CHB up to date. The results demonstrated that prematurely switching to single-drug maintenance therapy would be resulted in high risk viral relapses, but prolonged combination therapy was effective to maintain sustained response in CHB patients in a clinical setting. To some extent, our results presented a controversy regarding whether it is able to reduce or withdrawal drug from combination therapy. Which were also consistent with previously published clinical trial reports on combination therapy (15, 16).

According to new AASLD and EASL guidelines for management of CHB (23, 24), less potent antiviral agents such as LAM and ADV have not recommended for monotherapy of patients with CHB in many countries. However, because of limitations on health insurance systems, costs, or availability of drugs, LAM and ADV are still widely used in developing countries (25, 26). In China, it is estimated that more than 50% of patients with CHB are receiving LAM or ADV monotherapy. Thus combination therapy in optimized suboptimal responses to ADV monotherapy or rescuing treatment failure to LAM monotherapy would be common for a long period of time in future. Due to the lack of data on combination therapy, the time and procedure in which the physician have to stop combination therapy is widely concerned (27). Theatrically, switching to single drug therapy is possible, because viral titer has already been suppressed in very low levels, and this potential therapeutic strategy is supported by previous studies, showing that less potent antiviral agents also could effectively control virus replication in patients with low viral loads (28-30). However, in present study, sustained response rates (including viral and biochemical response rates) in patients who switched to single-drug maintenance therapy were significantly lower than patients who continued combination therapy. These findings suggest that only long-term combination therapy could ensure sustained efficacy in patients with CHB; in contrast, switching to single-drug maintenance therapy would result to high risk of viral relapse.

In this ‘real-world’ cohort, genotypic resistance mutations were not detected immediately after the emergence of viral rebound, hence all patients chose to continued current treatment during 3-month careful observation. After retrospective analysis of frozen serum samples that had been previously collected, we found that 3 patients in group B with single-drug maintenance therapy had NAs-resistance, and resistance rate was 15.8% at month 12 after changing to single-drug maintenance therapy. Thus, prematurely switching to single-drug maintenance therapy would not only result to viral relapse and resistance, but also can lead to the loss of achieved clinical benefits and risk of disease progressions.

Some studies reported the efficacy of antiviral therapy was different in patients with positive HBeAg and negative HBeAg (31, 32). Due to the small sample size, subgroup analysis of sustained efficacy based on HBeAg statue was not carried out in both groups. However, univariate analysis in present study showed that HBeAg positivity prior to combination therapy (LAM+ADV or LDT+ADV in optimizing ADV suboptimal resposne) was a significantly high risk factor of viral relapse in patients, who switched to single-drug maintenance therapy in this study.

In previous studies, lower baseline viral load and early viral response had been shown to be a good predictor of long-term sustained viral response and drug resistance (29, 33). In this study, we found that serum HBV DNA level at month 6 of past combination therapy were associated with probability of SVR in patients who switched to monotherapy. Of patients switched to single-drug maintenance therapy, detectable serum viral level at month 6 of past combination therapy before this study had a negative predictive value of 100% for SVR at month 12. This finding suggested that detectable serum viral level at month 6 after starting combination therapy was associated with an extremely lower probability of SVR after switching to single-drug maintenance therapy.

In this study, the majority of patients were well tolerated, and discontinuation due to adverse events was not observed. The overall safety profile of either combination therapy or single-drug maintenance therapy in CHB patients was similar to those reported in other studies (4, 32, 34).

Limitations of this prospective study are the small sample size and a potential bias in treatment assignment due to the study design, and clinical trials with large sample size and low risk of bias are needed to confirm our findings. However, statistical analysis showed that demographical and laboratory characteristics between two groups were comparable, which reduced the bias of treatment assignment in a certain extent.

In conclusion, prolonged combination therapy was effictive to maintain sustained viral responses in a ‘real-world’ setting.
